# Deep learning model for automatic differentiation of EMAP from AMD in macular atrophy

**DOI:** 10.1038/s41598-023-47854-7

**Published:** 2023-11-21

**Authors:** Maxime Chouraqui, Emanuele Crincoli, Alexandra Miere, Isabelle Anne Meunier, Eric H. Souied

**Affiliations:** 1 Department of Ophthalmology, Centre Hospitalier Intercommunal de Créteil, 40, Avenue de Verdun, 94100 Créteil, France; 2 Catholic University of “Sacro Cuore”, Rome, Italy; 3 National Reference Center for Inherited Sensory Diseases, University Hospital of Montpellier, University of Montpellier, Montpellier, France; 4 Sensgene Care Network, Strasbourg, France; 5 Institute for Neurosciences of Montpellier, Inserm, University of Montpellier, Montpellier, France

**Keywords:** Diseases, Medical research

## Abstract

To create a deep learning (DL) classifier pre-trained on fundus autofluorescence (FAF) images that can assist the clinician in distinguishing age-related geographic atrophy from extensive macular atrophy and pseudodrusen-like appearance (EMAP). Patients with complete outer retinal and retinal pigment epithelium atrophy secondary to either EMAP (EMAP Group) or to dry age related macular degeneration (AMD group) were retrospectively selected. Fovea-centered posterior pole (30° × 30°) and 55° × 55° degree-field-of-view FAF images of sufficiently high quality were collected and used to train two different deep learning (DL) classifiers based on ResNet-101 design. Testing was performed on a set of images coming from a different center. A total of 300 patients were recruited, 135 belonging to EMAP group and 165 belonging to AMD group. The 30° × 30° FAF based DL classifier showed a sensitivity of 84.6% and a specificity of 85.3% for the diagnosis of EMAP. The 55° × 55° FAF based DL classifier showed a sensitivity of 90% and a specificity of 84.6%, a performance that was significantly higher than that of the 30° × 30° classifer (*p* = 0.037). Artificial intelligence can accurately distinguish between atrophy caused by AMD or by EMAP on FAF images. Its performance are improved using wide field acquisitions.

## Introduction

Extensive macular atrophy and pseudodrusen-like appearance (EMAP) is a clinical entity affecting mostly women after 50 years old and characterized by the association of a central atrophy and pseudodrusen-like lesions. Macular atrophic lesions are usually symmetric and bilateral with a large vertical axis and peripheral fundus shows multiple areas of pavimentous degeneration. Coherently with the central and peripheral involvement, the main symptoms are central scotoma and nyctalopia^1^. The presence of central atrophy in the context of pseudodrusen lesions give way to frequent misdiagnosis of much more frequent conditions leading to geographic atrophy (GA), the most frequent of which is age related macular degeneration (AMD). Compared to AMD, EMAP is characterized by a faster evolution and earlier onset (around fifty years old). These factors, along with the bilateral central involvement and the peripheral degeneration affecting almost the entire field of view in both eyes, are responsible for a particularly unfortunate visual prognosis in patients diagnosed with EMAP. Moreover, EMAP patients normally do not develop macular neovascularization. In this perspective, it is important to perform early differential diagnosis between this condition and other diseases leading to GA in order to provide the patient with the best possible prognostic information. In the last years, deep learning (DL) models have shown their effectiveness in image-centric specialties, such as Ophthalmology. The application of DL models in Ophthalmology mostly focused on early diagnosis and staging of high prevalence sight-threatening diseases such as diabetic retinopathy (DR) or dry AMD based on color fundus photography (CFP) analysis^2^. A series of studies also evaluated the performance of DL classifiers based on fundus autofluorescence (FAF) imaging, proving it to effectively distinguish GA from other retinal diseases without atrophy such as AMD without GA, adult-onset foveamacular vitelliform dystrophy, central serous chorioretinopathy, and epiretinal membranes^3–5^. FAF is currently the most used enface method for evaluation of extension and characterization of GA in AMD and non-AMD patients, and it has also been demonstrated to provide information useful for the detection of fast progressing phenotypes in AMD^6,7^. The aim of our study is therefore to evaluate the possibility that a DL classifier based on FAF images can assist the clinician in distinguishing age-related GA from EMAP in order to improve the quality of prognostic information in these patients.

## Materials and methods

### Design of the study

**Figure 1:**
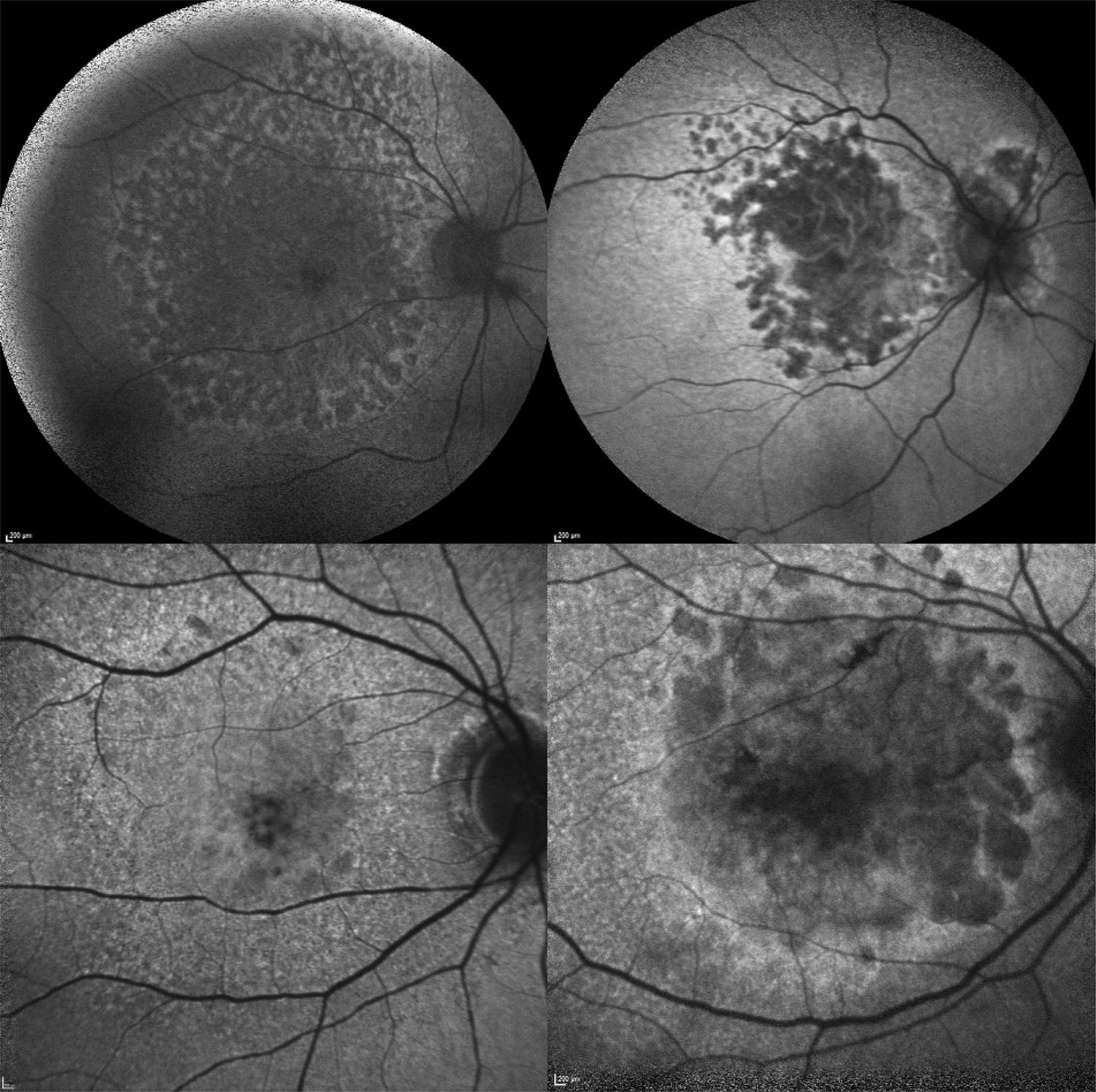
Example of 55° × 55° FAF images (first row) of a case of EMAP (upper left) and a case of GA from dry-AMD (upper right). Lower row shows examples of 33° × 33° FAF images from an EMAP (lower left) and a dry-AMD (lower right) case from different patients. AMD, age related macular degeneration; EMAP, extensive macular atrophy and pseudodrusen; FAF, fundus autofluorescence; GA, geographic atrophy.

A retrospective screening of patients diagnosed with EMAP or GA for AMD was conducted among patients referring to Hospital Intercommunal de Creteil and University Hospital of Montpellier between April 2007 and December 2021. Included patients presented complete outer retinal and RPE atrophy secondary to either EMAP (EMAP Group) or to dry AMD (AMD group). Confirmation of the diagnosis was provided by multimodal imaging evaluation (including fundus slit lamp examination, FAF and OCT B-scan) by two different senior retina specialists in Creteil Department of ophthalmology. In case of disagreement between the two graders, a third senior grader with extensive experience in the field of macular diseases was asked to deliberate final diagnosis. Only images from the most affected eye were considered and included in training, validation and testing of the model. The criteria for EMAP diagnosis were: polycyclic well-delineated chorioretinal atrophy extending to the temporal vascular arcades, with a larger vertical axis and without sparing of the fovea featured the macular lesion, pseudodrusens on the posterior pole and/or peripheral retina, paving stones on the peripheral was an argument in favor the diagnosis of EMAP however it was not necessary. Final diagnosis was used as a ground truth for training of the DL classifier. Patients with overlapping retinal diseases, low quality images (Q score < 15) and optic disk drusens were excluded from enrollment. A number of FAF acquisition ranging from a minimum of 2 to a maximum of 4 for each patient, was included in the DL process. The different number of acquisitions available for each patient was due to the retrospective nature of the study. FAF images were acquired with Spectralis HRA + OCT (Heidelberg Engineering, Heidelberg, Germany). All available posterior pole (30° × 30°) FAF acquisitions centered on the fovea were used to generate a first DL classifier. Available fovea centered 55° × 55° degree-field-of-view images were used to elaborate a second DL classifier (see Fig. 1). The study protocol was approved by the Institutional Ethics Committee and informed consent with complete explanation of the protocol was provided to all participants in accordance with the Declaration of Helsinki.

### Development of the deep learning classifier

The DL classifier was developed using MatLab software (Mathworks, Natick, MA) Deep learning toolbox. ResNet-101 (Microsoft ResNet; Microsoft Research Asia, Beijing, China) Convolutional Neural Network (CNN) pretrained with ImageNet dataset (http://www.image-net.org/) was used for the process. The high resolution FAF images (1536 × 1536 pixels) were rescaled to 224 × 224 pixels to meet the CNN specifications, with the fovea at the center. Eighty (80) % of the set of images from Hospital Intercommunal de Creteil was used for training while the remaining 20% of images from this set were randomly selected for validation. Augmentation techniques included rotation (from − 20° to + 90°) and spatial translation. Adam optimization algorithm was used to reduce loss. Regularization was obtained by a combination of drop out and weight constrain techniques. Testing was performed on an external set of images (corresponding to 20% of the sample size of Creteil set) coming from University Hospital of Montpellier Ophthalmology Center. The number of images used for testing corresponded to the 20% of the total amount. The same procedure far performed twice: at first using only 30° × 30° images and secondarily using only 55° × 55° images.

### Statistical analysis

SPSS software was used for the analysis. Differences in terms of age and sex between patients were evaluated using two-tailed T test for independent samples and Chi^2^ test respectively. Normality of the distribution for age was evaluated using Shapiro–Wilk test. Performance of the two DL classifiers were evaluated through a comparison of the CNN output to the ground truth (clinical diagnosis by expert graders). Sensibility, specificity, positive predictive value (PPV) and negative predictive value (NPV) as well as Area Under the Receiver Operating Characteristics (AUROC) curves for the two were reported. AUROCs were compared using DeLong method. A *p* value < 5% was considered as statistically significant.

### Ethics approval

All procedures performed in studies involving human participants were in accordance with the ethical standards of the institutional and/or national research committee and with the declaration of Helsinki and its later amendments or comparable ethical standards. The study was approved by the Ethics Committee of the Federation France Macula in 2021.

### Informed consent

Informed consent was obtained from all individual participants included in the study.

## Results

**Figure 2:**
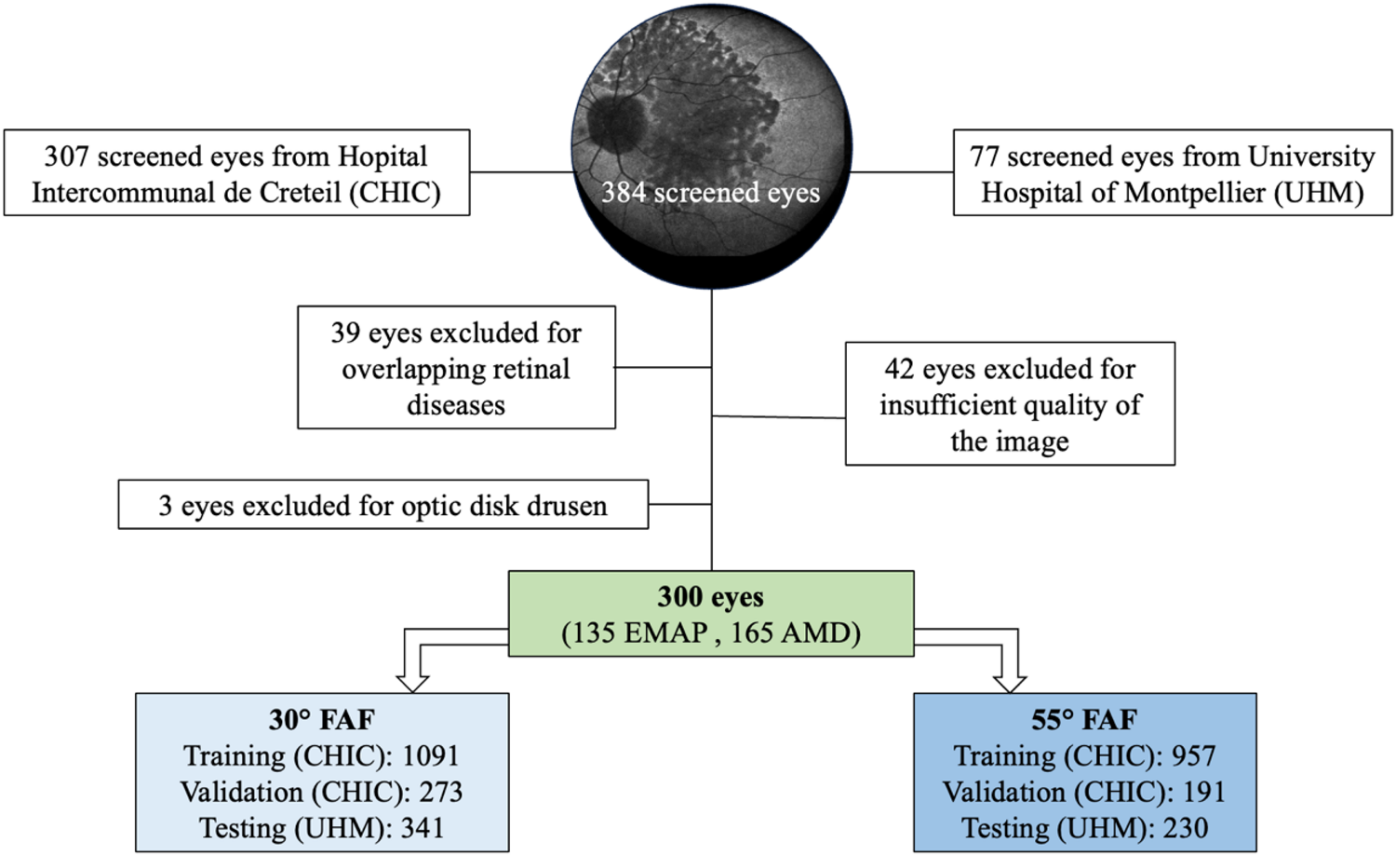
Flowchart illustrating the selection process of the study population. The number of images used for the development of the DL models is also reported. CHIC, Hopital Intercommunal de Creteil; DL, deep learning; UHM, university hospital of Montpellier.

A total of 300 patients were recruited, 135 belonging to EMAP group and 165 belonging to AMD group. Mean age of the population was 71.3 ± 5.6 years in the EMAP group and 73.1 ± 7.2 years in AMD group (*p* > 0.05). Male patients were 61/135 (45.19%) in EMAP group and 79/165 (47.87%) in AMD group (*p* > 0.05). A flowchart illustrating the selection process of the study population is illustrated in Fig. 2.

The 30° × 30° FAF based DL classifier was trained on 1091 images, validated on 273 and tested on 341 images. Among the 341 testing images, 211 belonged to patients from AMD group and 130 to patients from the EMAP group. The DL classifier correctly identified 110 cases over 130 of EMAP and 180 cases over 211 of AMD, showing a sensitivity of 84.6% and a specificity of 85.3% for the diagnosis of EMAP. The positive predictive value (PPV) was 78.0% and the negative predictive value (NPV) of 90.0% while the AUROC was 0.851 (CI 0.827–0.869). Confusion matrix describing the 30° × 30° DL classifier performance on EMAP cases is reported in Table 1.

**Table 1:** Confusion matrix describing performances of the 30° × 30° FAF based DL classifier on detection of EMAP cases.

30° × 30° FAF		CNN	
		Positive	Negative
Ground truth	Positive	110	20
	Negative	31	180

CNN, convolutional neural network; EMAP, extensive macular atrophy and pseudodrusen-like appearance; FAF, fundus autofluorescence.

**Figure 3:**
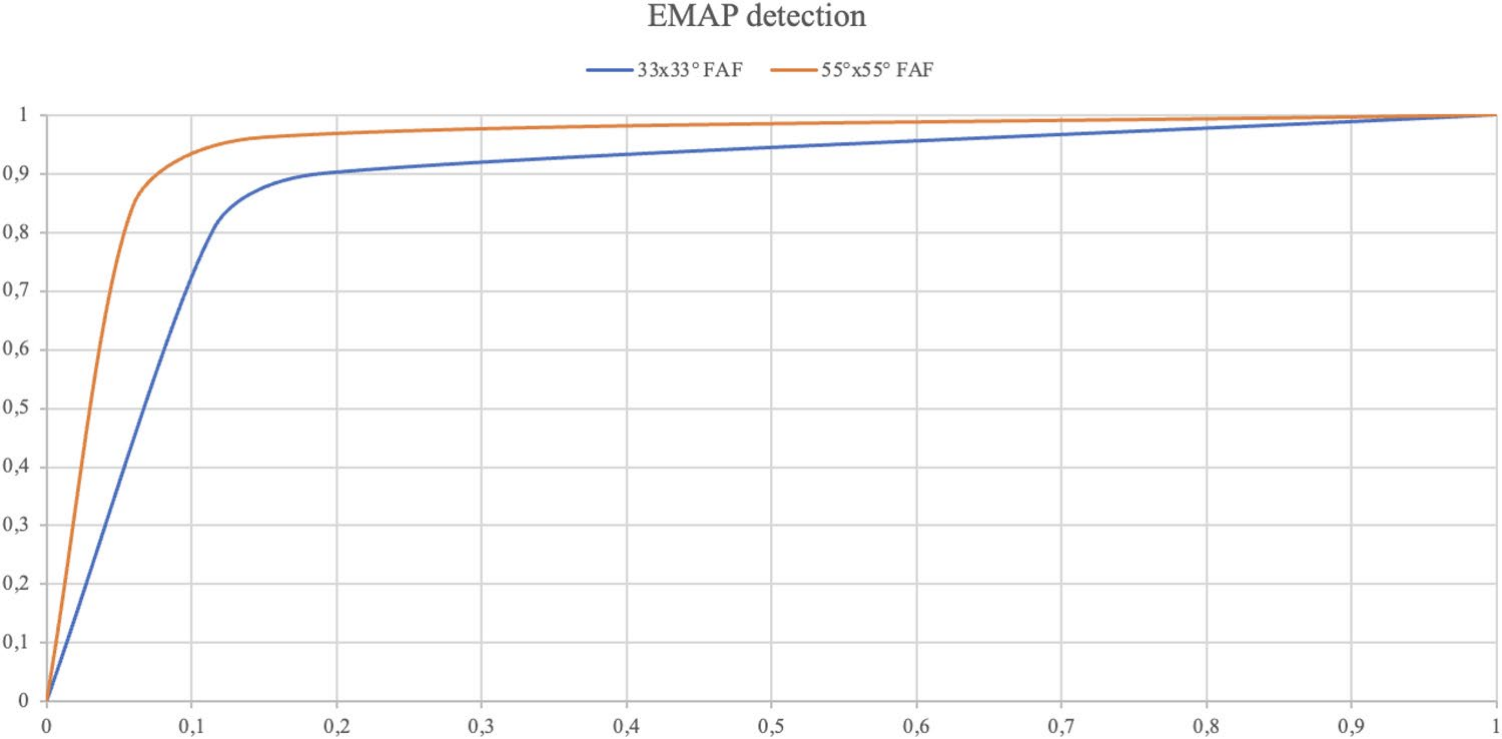
AUROCs for 33° × 33° FAF image-based DL classifier (blue curve) and 55° × 55° FAF images-based DL classifier (orange curve). The difference between the two was statistically significant according to DeLong method (*p* = 0.037). DL, deep learning; EMAP, Extensive macular atrophy and pseudodrusen-like appearance; FAF, fundus autofluorescence.

The 55° × 55° FAF based DL classifier was trained on 957 images, validated on 191 and tested on 230 images. Among the 230 testing images, 130 belonged to patients from AMD group and 100 to patients from the EMAP group. The DL classifier correctly identified 90 cases over 100 of EMAP and 110 cases over 130 of AMD, showing a sensitivity of 90% and a specificity of 84.6% for the diagnosis of EMAP. The positive predictive value (PPV) was 81.8% and the negative predictive value (NPV) of 91.6% while the AUROC was 0.882 (CI 0.860–0.899). Results of the 55° × 55° FAF based DL classifier are reported in the confusion matrix at Table 2. There was a statistically significant difference between AUROC from 30° × 30° DL classifer and AUROC from 55° × 55° DL classifier (*p* = 0.037) (see Fig. 3).

**Table 2:** Confusion matrix describing performances of the 55° × 55° FAF based DL classifier on detection of EMAP cases.

55° × 55° FAF		CNN	
		Positive	Negative
Ground truth	Positive	90	10
	Negative	20	110

CNN, convolutional neural network; EMAP, extensive macular atrophy and pseudodrusen-like appearance; FAF, fundus autofluorescence.

## Discussion

GA is responsible for 20% of cases of legal blindness in AMD patients. GA areas are visible on fundus examination as sharply demarcated hypopigmented areas, in which larger choroidal vessels may become visible owing to the absence of the RPE and the choriocapillaris. In AMD related GA, peripheral extension of the atrophy is typically faster and patients maintain good central vision until foveal involvement, which occurs at a later stage. Histopathologic investigation of these areas showed cell death in the RPE, outer neurosensory retina, and choriocapillaris and modern in vivo technology allowed confirmation of these pathological elements. SD-OCT shows choroidal signal enhancement (due to loss of absorbing pigment) and thinning and disruption of the outer retinal layers, including the outer nuclear layer. Atrophy of the RPE and loss of fluorophores induce complete loss of autofluorescence signal in these areas at FAF imaging, which is accompanied by a variable pattern of hyper autofluorescence in adjacent areas^8^. In 2009, Hamel et al.^1^ described a cohort of 18 patients in their 5th decade (age range 41–54) showing a specific GA phenotype (which is now known by the name of EMAP) characterized by peripheral degeneration symptomatic for difficulties in dark adaptation and later onset of central scotoma in the context of macular atrophic degeneration. Subtle differences may be noticed in atrophy shape: in AMD, atrophy is circular, centered on the fovea with a larger horizontal axis whereas in EMAP atrophy is oval, polycyclic with a larger vertical axis^1^. Differently from AMD patients these patients do not develop choroidal neovascularization and are instead characterized by peripheral retina disfunctions. Since peripheral symptoms such as dark adaptation impairment and visual field reduction often lie unnoticed to patients’ awareness until late stages, the first symptom leading to medical referral in EMAP patients is often the appearance of central scotoma. Given the presence of GA at the posterior pole, these patients are likely to be misdiagnosed with the much more prevalent GA related to AMD, since subtle differences in sex, age and inflammatory profile are often not sufficient to raise suspect of an EMAP case. In fact, small series have shown that EMAP seem to most frequently affect women and is associated to eosinophilia, lymphocytosis, increased erythrocyte sedimentation rate, decreased CH50, and high plasma C3 level which may suggest an association between EMAP and a systemic inflammatory profile^9^. By contrast, other authors didn’t detect a blood inflammatory profile in EMAP patients^10^. Interestingly, in our large cohort, no significant difference in sex prevalence and age was noted between AMD and EMAP patients. An additional element of confusion is given by the fact that EMAP cases are characterized by the presence of pseudodrusen and often show a “diffuse-trickling” FAF pattern, which is typically related to faster progressing GA phenotypes in AMD^10^. Altogether, these elements account for a high risk of mismanagement of EMAP cases diagnosed as AMD cases. Despite the presence of a number of studies^3,4,11,12^ detecting and classifying GA with deep learning methods, our study is the first in literature to show results of an automatic differential diagnosis between GA caused by AMD and EMAP cases. Our findings reveal good performance of the FAF imaging based deep learning classifier presented in the study, showing a sensitivity of 84.6% and a specificity of 85.3% for the diagnosis of EMAP on 30° × 30° FAF images and a sensitivity of 90% and a specificity of 84.6% for the diagnosis of EMAP on 55° × 55° FAF images. Interestingly, wide field images allow a significantly higher sensitivity of the DL classifier, suggesting peripheral alterations in the FAF pattern that still lay unnoticed to human eye detection. A FAF imaging-based DL classifier has recently been evaluated for the distinction between GA from AMD and atrophy due to STGD1 and Pseudo-Stargardt multifocal pattern dystrophy. This model achieved a training accuracy of 0.89 on the test set with the model training with 100 epochs and 0.92 using 10 epochs and an AUC-ROC of 0.981^4^. Besides FAF, OCTA could help us to differentiate GA from EMAP with specific defect. Indeed Rajabian et al. found three retinal regions in EMAP disease corresponding to progressively deeper perfusion defects. Quantitative analyses of retinal vessels revealed significant alterations, especially in the DCP and CC, in both atrophic and junctional zones in retina of EMAP patients compared with preserved zones and controls^13^.

Among the strengths of the study, it should be mentioned the validation of the DL classifier on an external set of images and thus the multicentricity of the investigation. Limitations of the study include the lack of comparison with DL methods based on different enface images or B scan methods and the lack of inflammatory profile testing in the study population.

In conclusion, our model could provide an automatic easy to use tool for differentiation of EMAP from early onset AMD in young patients showing macular atrophy. This could allow correct clinical management from the earliest phases, representing a valid tool in the setting of personalized medicine.

## Data Availability

The datasets used and/or analysed during the current study are available from the corresponding author on reasonable request and with permission of Centre Hospitalier Intercommunal de Creteil.
